# Supported housing programs for persons with serious mental illness in rural northern communities: A mixed method evaluation

**DOI:** 10.1186/1472-6963-8-156

**Published:** 2008-07-24

**Authors:** Phyllis Montgomery, Cheryl Forchuk, Craig Duncan, Don Rose, Patricia H Bailey, Ramamohan Veluri

**Affiliations:** 1 School of Nursing, Laurentian University, Ramsey Lake Road, Sudbury, Ontario, P3E 2C6, Canada; 2 Faculty of Health Sciences, Lawson Health Research Institute, University of Western Ontario, 1151 Richmond Street, Suite 2, Health Sciences Addition, H38 London, Ontario, N6A5C1, Canada; 3 Daphne Cockwell School of Nursing, Ryerson University, 350 Victoria Street, Toronto, Ontario, M5B 2K3, Canada; 4 Department of Psychiatry, University of Western Ontario and Northern Ontario School of Medicine 680 Kirkwood Drive, Sudbury, Ontario, P3E 1X3, Canada

## Abstract

**Background:**

During the past two decades, consumers, providers and policy makers have recognized the role of supported housing intervention for persons diagnosed with serious mental illness (SMI) to be able to live independently in the community. Much of supported housing research to date, however, has been conducted in large urban centers rather than northern and rural communities. Northern conditional and contextual issues such as rural poverty, lack of accessible mental health services, small or non-existing housing markets, lack of a continuum of support or housing services, and in some communities, a poor quality of housing challenge the viability of effective supported housing services. The current research proposal aims to describe and evaluate the processes and outcomes of supported housing programs for persons living with SMI in northern and rural communities from the perspective of clients, their families, and community providers.

**Methods:**

This research will use a mixed method design guided by participatory action research. The study will be conducted over two years, in four stages. Stage I will involve setting up the research in each of the four northern sites. In Stage II a descriptive cross-sectional survey will be used to obtain information about the three client outcomes: housing history, quality of life and housing preference. In Stage III two participatory action strategies, focus groups and photo-voice, will be used to explore perceptions of supported housing services. In the last stage findings from the study will be re-presented to the participants, as well as other key community individuals in order to translate them into policy.

**Conclusion:**

Supported housing intervention is a core feature of mental health care, and it requires evaluation. The lack of research in northern and rural SMI populations heightens the relevance of research findings for health service planning. The inclusion of multiple stakeholder groups, using a variety of data collection approaches, contributes to a comprehensive, systems-level examination of supported housing in smaller communities. It is anticipated that the study's findings will not only have utility across Ontario, but also Canada.

## Background

Homelessness is a major health-related problem in Canada [1,2]. The number of homeless individuals reported ranges from 14,000 [3], "tens of thousands" [4]. Research also shows homelessness is more prevalent amongst persons living with severe mental illness (SMI) than in the Canadian population at large [1,4-6]. Kirby and Keon [7] suggest that approximately 30% to 40% of homeless people have mental health problems. Of those, as many as 25% also have an addiction problem. Such statistics, however, cannot begin to speak to the stigma and discrimination that persons with SMI encounter while trying to secure safe and adequate housing.

An intervention to prevent homelessness for people living with SMI is the supported housing approach [8]. This approach values the interplay of client choice, community integration, and flexible support with regard to housing. Emphasis on normal housing, work and social networks requires the implementation of individualized and flexible care processes delineated by the clients' goals and preferences [1,9,10]. Consumers, providers and policy makers recognize the effectiveness of supported housing for realizing positive health outcomes. According to Forchuk, Ward-Griffin, Csiernik and Turner [11], supported housing for homeless persons with mental illness allows for connections with significant others in addition to providing a sense of safety and purposefulness. Yet little is known about what supported housing elements, individually or in combination, are most significant for patient success [12,13].

To date, supported housing research has been conducted primarily in urban settings, focusing on indicators such as service utilization, housing stability, and other financial measures [1,12]. Until the late 1990s much of the supported housing research being conducted was descriptive and focused on consumer characteristics and outcomes [14-16]. Rog [17] reviewed the research evidence regarding the effects of supported housing on patient outcomes. He found that existing research, divergent as it is, strongly suggests that persons with SMI can live successfully in a range of housing types. Supporting this view are other Canadian researchers [11,18-20]. What remains unknown is the effect of supported housing intervention on outcomes for rural clients [21].

Researchers have also examined mitigating variables such as consumers' preferences for housing and support [22-25]. A shared finding is that consumers preferred to live in their own place, either alone or with a significant other rather than with other mental health consumers. Goldman et al. [22], however, suggests a cautious interpretation of such a finding since prior studies used professional- rather than client-designed measures of consumer preference.

Although limited, available evidence suggests housing with supports has positive effects on the clients' quality of life. Matching consumers' needs to specific services is the most cost-effective approach [26,27]. Housing combined with appropriate supports stabilizes the lives of persons characterized as chronically homeless. For example, a one-year study by Clark and Rich [28]examined the impact of supported housing and case management on measures of housing status, mental health symptoms, substance use, physical health and quality of life. The researchers found that persons with high psychiatric symptom severity achieved a higher quality of life with the supported housing program than with case management alone. As well, they reported that persons with low and medium levels of psychiatric symptoms did just as well as those with case management alone. These findings reinforce the importance of matching service type to clients' needs rather than delivering a prescriptive program; an emphasis challenged by northern and rural communities' lack of appropriate, accessible supportive services.

The success of the supported housing intervention is influenced by the characteristics, circumstances and resources of each community. As Lamb and Bachrach [29]state, "there is no single kind of housing that can effectively meet the needs of all long-term mental health patients" (p. 1043). Persons with SMI require a range of housing and support options. Salient policy documents such as *Respecting Housing and Support *[30] and *Out of the Shadows at Last *[7], assert that enabling people with mental illness to live safely in the community requires three interconnected elements. These include more housing units, more assistance so that people can afford existing units, and more supportive services. The interplay among these three factors is by no means prescriptive, thereby reinforcing the "non-cookie cutter" feature of implementing supported housing with a person coping with SMI.

Implementation of housing with supports in northern and rural communities is further confronted by factors such as small or non-existent housing markets, "aging" demographic trends, rural poverty, quality of housing, lack of mental health care services, lack of a continuum of housing services and economic and labor force changes [31,32]. Smaller communities have housing issues that often receive little attention in regards to policy [33]. As well, geography, population density, and the availability of mental health services offer unique challenges to evaluation research in northern and rural communities. Nevertheless, literature examining the methodological issues associated with assessing community mental health programs suggests a new role for evaluation in community mental health [8,34,35].

### Objective and aims

The authors of this study contend that evaluation research fosters insight not only on how inquiry constructs knowledge but on how we create "formative narratives" for change. Despite the available evidence about the effects of supported housing, it is still unclear what elements within a particular supported housing approach and environmental context (rural setting) facilitate effective service provision for persons with SMI. Researchers [21,32,33] recommend further study on the processes of supported housing programs to identify the key elements of effective rural housing programs and their relationship to outcomes such as mental status, social functioning and quality of life. In addition, more research exploring the significance of these elements from the perspectives of consumers, families and service providers is needed [12,14,17].

The overall objective of this research is to describe and evaluate the processes and outcomes of supported housing programs for persons living with a serious mental illness (SMI) in northeastern Ontario from the perspective of clients, their families and community workers. The research questions guiding this inquiry are as follows:

1. What are clients' quality of life, housing stability, and housing preferences?

2. For clients residing in four Northeastern communities, what differences occur in their quality of life, housing stability, and housing preference?

3. What are the differences between Northeastern clients' quality of life, housing stability, and housing preferences and a Southwestern comparison group drawn from a Community University Research Alliance (CURA) sample from London, Ontario?

4. What are clients'/families'/providers' perceptions of the elements of effective supported housing programs?

5. What supported housing services need to be changed in order to make the most difference in the day-to-day lives of clients?

This study will also generate hypotheses for future research.

### Theoretical Framework

**Figure 1:**
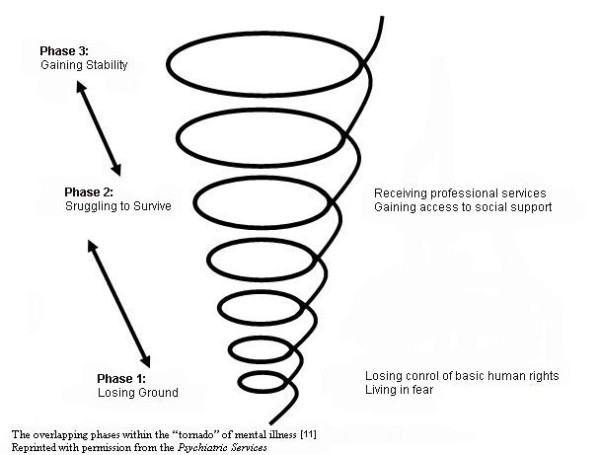
The overlapping phases within the "tornado" of mental illness (Forchuk et al, 2006).

This research will be guided by Forchuk, Ward-Griffin and Turner [11]conceptualization of *Getting, Losing and Keeping Housing *(Figure 1), originating from the housing experiences of 90 psychiatric consumers living in urban and rural areas in southwest Ontario. Their housing experiences involved three phases: losing ground related to limited control over their basic human rights and inappropriate housing conditions; struggling to survive with the support of various community services; and gaining stability as a result of securing personal space and rebuiding relationships. The model illustrates overlapping boundaries between processes and outcomes associated with housing. For example, achievement of housing stability requires accessing and receiving support services. To further understand the processes of securing housing and its outcomes such as quality of life, housing stability and housing preference, this conceptualization emphasizes listening to multiple perspectives (clients, families, and providers).

## Methods

### Design

**Figure 2:**
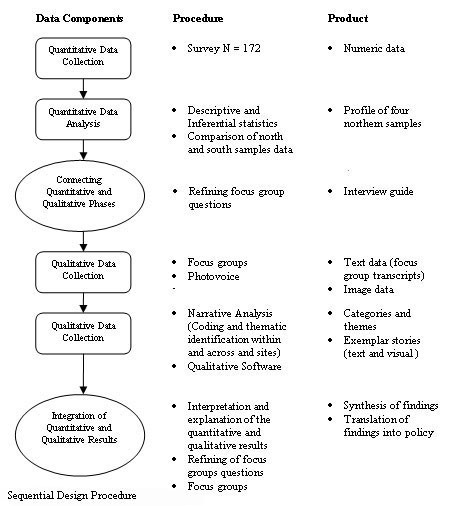
Sequential design procedure.

**Figure 3:**
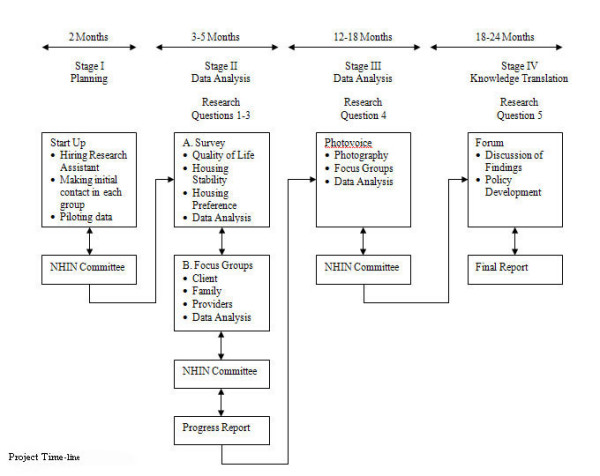
Project time-line.

The study will use a mixed-methods design involving quantitative and qualitative methods that will be informed by participatory action research (PAR) (Figure 2). The combination of quantitative and qualitative methods will allow a more robust analysis [36] and provide multidimensional answers of maximum relevance to the research questions [37-39]. Quantitative data will provide baseline data related to sample characteristics, quality of life, housing stability and housing preference. The qualitative data will assist the researchers in further exploring the quantitative findings in relation to complex outcome variables such as quality of life [40]. Blending these approaches will allow for the findings to be considered within the context of perspectives of clients' and supported housing service providers. The study will be completed over a two-year period and will involve an iterative process in four sequential stages: planning, two stages of data gathering, and knowledge synthesis and translation. Figure 3 illustrates more specifically how the project will be conducted in each phase. All phases will be conducted in consultation with an existing advisory committee, the Northern Homelessness Initiatives Network (NHIN): a committee established in 2000 for the purpose of creating, supporting, and sharing knowledge among housing services for persons with SMI. Their mandate is to build positive, professional relationships among supported housing agencies for people diagnosed with SMI who are living in northern Ontario. In preparation for this study, the network supported the pilot testing of photo-voice (a method detailed in Stage III below) in three housing programs.

### Setting

The setting for this research is northeastern Ontario, a geographical area covering over 276,000 square kilometers. In each of the four districts, there is a variety of non-profit service or sets of services offering supported housing. Housing, scattered though each of the four communities, is not dependent on accepting support services, and the range of housing types varies across the four communities. Variation in supported housing implementation across the four communities is attributed to funding, availability and qualifications of service providers; knowledge uptake barriers; culture; and geography. Combined, over 500 persons with SMI are either receiving housing with supports or waiting to access such services. A shared program goal of all services is to assist persons with a SMI to integrate into the community by maintaining or improving a person's psychiatric functioning, independent living skills, and housing stability. This goal is consistent with both the elements and notions of preventing the "tornado" of mental illness [11]. Another shared feature is that the supported housing programs either operate under the mandate of the Canadian Mental Health Association, or the CMHA is at least involved with these programs and the agencies that administer them. Furthermore, each CMHA has an Executive Director who is a member of the NHIN, and all of these agencies will participate in the study.

### Procedure

#### Stage I

This stage is particularly crucial as formal research has not been conducted in two of the four sites. Members of the research team, in consultation with the Executive Director and an identified research partner at each site, will collaborate concerning strategies to introduce the research to the supported housing service(s). Other activities include hiring a project coordinator and site data collectors; training research staff for data collection, data entry into software programs (SPSS and NVivo); meeting with NHIN; and securing ethical approval at each site (Figure 3).

#### Stage II

Stage II will address the first three research questions related to clients' quality of life, housing stability, and housing preference. A descriptive cross-sectional survey design and quota sampling will be used. To be eligible for inclusion in this study, participants must using or waiting for supported housing services, understand English, and be willing to provide informed consent. To be eligible for CMHA housing services, a client must have a SMI as defined by diagnosis, duration and disability [41]. A minimum sample size is 43 persons per site using the standard deviations and mean scores from the CURA data related to the Lehman Quality of Life scale with a power of .80 and an alpha of .05.

Data will be collected using four self-report survey tools: Demographic Profile; Lehman's Quality of Life Interview-Brief Version (LQOLI-BV) [42], Housing History Survey [43], and Consumer Housing Preference Survey Short Version (CHPSSV) [44]. The Demographic Profile consists of 10 items. The LQOLI measures clients' objective quality of life experiences and subjective feelings about these experiences in eight domains: residency, daily functioning, family relations, social relations, leisure activities, finances, safety and legal problems, work and school, and health. It is a structured self-report interview and takes approximately 30–45 minutes to complete. The brief version of the LQOLI to be used is reportedly easier to administer since clients are often more receptive to answering fewer questions. When used for individuals with chronic mental illnesses, the reported internal consistency of the LQOLI's various subscales, range from .86 to .90. The Housing History Survey was developed through a CURA on mental health and housing [43]. The form asks participants to list all their places of residency in the last two years, their duration at each residence, and whether the move was desirable. As well, they are asked to rate the housing on a 7-point satisfaction scale, 1 (delighted) to 7 (terrible). The fourth measure, the CHPSSV, was developed at the Centre for Community Change through Housing and Support (now known as the Centre for Community Change International). This is the same institute that pioneered Carling's notion supported housing [44]. The CHPSSV contains 22 questions about demographic information, current housing, preferred housing, preferred living companions and supports needed. All instruments will be administered by a trained researcher member in each site.

The study will be introduced to prospective client participants by familiar community mental health workers. Interested participants will be approached by a trained research member situated in each of the four sites. Informed consent will be obtained from all participants. During the consent process, participants will be informed that they may be contacted by the site's research member about participating in Stage III of the research study. Signed consent forms will be returned to the study's Project Coordinator for storage in a locked cabinet. Incentives for clients to participate will include strategies such as: transportation, forwarding of reminder letters for those with shared phone lines or no phones, and offering a $20.00 (Canadian) reimbursement.

Descriptive statistics will be used to describe the sample's characteristics and their quality of life, housing stability and housing preference. Results of the analysis will serve as the basis for discussion in the project's subsequent stages. A correlation matrix will examine the relationship between all major variables and selected demographic characteristics. Inferential statistics will be used to examine the differences and association among the four northern sites. The northern data will be matched to a comparison group drawn from a Community University Research Alliance (CURA) sample from London, Ontario. *T*-tests will be used to examine outcome differences between these groups.

#### Stage III

A participatory action method will explore research question four concerning clients', families', and providers' perceptions of supported housing in each community. Data will be collected through photo-voice and focus groups.

Photo-voice, a visual method, involves the use of cameras by participants. According to [45], the three goals of this strategy are: to enable individuals to record and reflect upon their everyday experiences; to promote discussion about their visual representations; and to heighten insight of the wider community including policy makers. This method has been shown to be effective for engaging vulnerable populations in research [46,47]. Quota sampling will be used to ensure that the resident sample is representative of the survey variables measured in Stage II. Eight clients from each site will be invited to participate in this stage of the study. Participants will be asked to take a minimum of 10 images addressing the following questions: Can you tell me a story about receiving housing services? What are the most important aspects of housing services for your housing stability? What are your concerns about the supported housing service? How might the supported housing services that you receive be improved? A focus group will be conducted at each site to allow participants to share and explain their photographs. The photographs will act as a visual cue for discussion about their supported housing experiences. It is anticipated that the focus group will run for less than two hours. Each focus group will be audiotaped and the stories relating the pictures to supported housing will be transcribed into text for the purpose of analysis.

Data will be entered into a software program. A narrative/story analysis process will be used to analyze the participants' interviews [48,49]. The underlying premise utilizing this analysis strategy is the recognition that individuals most effectively make sense of their everyday circumstances by telling stories [50]. This systematic story analysis process will help the researchers identify the processes of the supported housing programs that relate to quality of life, housing stability and housing preference within and across sites.

Focus groups [51] will be used to collect data from the perspectives of clients, their families and community mental health workers about their local supported housing program. Purposive sampling will be used to identify clients who are 18 years of age or older, family members and community mental health workers for separate focus groups. Focus group members will be recruited through announcements about the study posted in key locations such as libraries, survivor programs, housing services and psychiatric outpatient services. All potential participants who self-identify and meet the above-stated inclusion criteria will be contacted by the site research member, provided with more information about the study, and invited to participate. Incentives for participation will be included. The following questions for the focus groups will be discussed: What is the perception of receiving housing services? What are the aspects of the housing service that are most important and useful for housing stability? What are problems with the current approach? How can it be improved?

The size of each of the three focus groups will be eight to 10 participants. Each focus group will meet face-to-face once and the session will be facilitated by an experienced member of the research team. Another research member will take detailed notes and manage the process' mechanics, such as audio taping the session. It is anticipated that the focus group will run for approximately 1 1/2 to 2 hours. Food will be served at the focus group. Each focus group will be audio taped and transcribed into text for analysis purposes. The method of focus group data analysis will be the same process as used in photo-voice, narrative/story analysis.

#### Stage IV

Stage IV will address the remaining research question: What supported housing services need to be changed in order to make the most difference in the day-to-day lives of clients? In a one day forum involving clients, families, community workers, community mental health groups, mental health decision makers and politicians, focus group methodology will be used to translate the findings into meaningful policy. In preparation for the forum, the researchers, using a sequential explanatory strategy created by [52] (Figure 3) will synthesize the findings from stages II and III. A mixed group of participants will be asked to review current policies related to the study's findings and then they will be asked to discuss the perspectives to the following questions: What current policies are associated with the key findings? What policies are successful in preventing housing "tornados"? What changes are feasible? What new policies need to be developed and implemented? What support is needed for new policies to be effective? As above, each focus group will be audiotaped and transcribed into text for the purpose of analysis. Data analysis will be the narrative/story approach.

## Discussion

This project is relevant at a local and provincial level. There has been no evaluation of the supported housing programs for persons with SMI in the four Northeast Ontario communities participating in this project. At a provincial level, the lack of similar studies in rural SMI populations speaks to its originality. Involving the NHIN committee from the inception of this initiative resulted in a systems-level evaluation and an inclusion of a variety of data collection strategies. The project is also novel in that it will address a significant knowledge gap in the delivery of supported housing services in non-urban settings. It focus on the long-term needs of persons with SMI will contribute to understanding effective strategies within the boarder supported housing intervention.

## Competing interests

The authors declare that they have no competing interests.

## Authors' contributions

All authors were involved in the writing and approving of the final manuscript.

## Pre-publication history

The pre-publication history for this paper can be accessed here:


